# Prediction values of tertiary lymphoid structures in the prognosis of patients with left- and right-sided colon cancer: a multicenter propensity score-matched study

**DOI:** 10.1097/JS9.0000000000000483

**Published:** 2023-05-26

**Authors:** Yonghuan Mao, Xingzhou Wang, Ling Xi, Meng Dong, Peng Song, Ji Miao, Cheng Lu, Sizheng Sun, Qiang Li, Chunzhao Yu, Xiaofei Shen

**Affiliations:** a Department of General Surgery, Nanjing Drum Tower Hospital Clinical College of Nanjing Medical University; b Department of General Surgery; c Department of Pathology, Nanjing Drum Tower Hospital, the Affiliated Hospital of Nanjing University Medical School; d Department of Gerontology, Geriatric Hospital of Nanjing Medical University; e Department of General Surgery, the Second Affiliated Hospital of Nanjing Medical University; f Department of General Surgery, Sir Run Run Hospital of Nanjing Medical University, Nanjing, China

**Keywords:** left-sided colon cancer, nomogram, prognosis, right-sided colon cancer, tertiary lymphoid structures, tumor budding

## Abstract

**Background:**

Tertiary lymphoid structures (TLS) are the lymphocyte aggregates that play a key role in the vast majority of solid tumors including colon cancer, displaying an antitumor effect under most circumstances. The heterogeneity between left- and right-sided colon cancer (LCC and RCC) encompasses various aspects, such as clinical manifestations, pathological features, and immune responses. However, the function and prognostic significance of TLS within LCC and RCC have yet to be fully understood.

**Methods:**

A retrospective analysis was performed on 2612 patients who underwent radical resection for LCC or RCC without distant metastasis in multiple medical centers. Utilizing propensity score matching, 121 patients with LCC and 121 patients with RCC were selected for the training set. An external validation set including 64 patients with LCC and 64 patients with RCC were also employed. Hematoxylin-eosin and immunohistochemical staining were used to assess TLS and the proportion of various immune cells. Clinical characteristics and prognostic values of TLS in patients with LCC and RCC were analyzed. Nomograms were constructed for LCC and RCC to predict 3-year and 5-year overall survival (OS), respectively.

**Results:**

For LCC and RCC patients, TLS was located in the interstitial region or outside the tumor tissue and mainly consisted of B cells and T cells. The TLS quantity and density in RCC were higher than those of LCC. In multivariate Cox regression analysis, TLS density (*P*=0.014), vascular invasion (*P*=0.019), and AJCC stage (*P*=0.026) were independent prognostic factors for 5-year OS of RCC. For LCC patients, AJCC stage (*P*=0.024), tumor differentiation (*P*=0.001), and tumor budding (*P*=0.040) emerged as independent prognostic factors for 5-year OS. Similar results were obtained in the external verification set. Separate nomograms for RCC and LCC were developed, displaying improved prediction performance compared to the AJCC 8th edition TNM staging system.

**Conclusions:**

Differences in TLS quantity and density were observed between LCC and RCC, suggesting that a nomogram based on TLS density could more effectively predict survival for RCC patients. Furthermore, a nomogram based on tumor budding was recommended for better prediction of LCC patient survival. Taken together, these results suggested that the immune and clinical characteristics of colon cancer at left and right side were substantially different, which may lead to the use of different prediction model and the development of individual treatment strategy.

## Introduction

Highlights

- Tertiary lymphoid structures number and density of right-sided colon cancer (RCC) were significantly higher than left-sided colon cancer (LCC).
- Tertiary lymphoid structures density, vascular invasion, and AJCC stage were independent prognostic factors for RCC.
- AJCC stage, tumor differentiation, and tumor budding were independent prognostic factors for LCC.
- A predictive model for the 3- and 5-year overall survival of LCC/RCC was constructed.

In recent years, the incidence of colorectal cancer in the whole world has continuously increased, seriously threatening the health of global people^1^. Currently, the prognosis of patients is generally predicted by evaluating tumor size, lymph node metastasis, and distant metastasis (AJCC eighth stage)^2, 3^. In 1990, Bufill *et al.* described colon cancer according to different anatomical sites. Subsequent reports pointed out that there were significant differences between left-sided colon cancer (LCC) and right-sided colon cancer (RCC) in embryonic origin, anatomical location, physiological effects, pathogenesis, pathological types, clinical manifestations, and molecular biology^4–7^. However, the roles and characteristics of immune responses in these cancers remain largely unexplored. Given the varying treatment outcomes for LCC and RCC^8,9^, an urgent need for the understanding of LCC and RCC including immune aspects should be taken into consideration to better clarify differences between RCC and LCC, which may guide future development of different treatment strategies and the development of a new effective prognosis prediction model.

Tertiary lymphoid structures (TLS) are ectopic lymphoid structures found in nonlymphoid tissues, such as tumors and chronic infections. They are aggregates of immune cells without a capsule surface, which may lead to faster migration of T cells into tumor tissues to exert their antitumor effects^10,11^. TLS has been shown to be a protective factor for predicting the prognosis of malignant tumors such as breast cancer, lung cancer, colorectal cancer, and pancreatic cancer^12–18^. In addition, TLS was suggested to be a site for generating memory T cells and B cells, which can be used as a prognostic indicator for postoperative patients with nonmetastatic colorectal cancer^19, 20^. However, contradictory results were also identified in liver cancer, where the presence of TLS was closely associated with a poor prognosis^21^. In addition, one study also showed that TLS density had no prognostic value in patients with stage III colon cancer^22^. Therefore, whether TLS could be used as a prediction factor in colon cancer needs to be better clarified, particularly given the aforementioned differences between RCC and LCC.

This study mainly analyzed relevant clinical parameters of patients with LCC and RCC, and explored the density, composition, and relationship between TLS and tumor-infiltrating immune cells (TILs) in patients with LCC and RCC. The significance of TLS density in guiding prognosis in patients with LCC and RCC was also analyzed, and prediction nomograms to predict the prognosis of LCC and RCC were also generated.

## Methods

### Patient population

**Figure 1:**
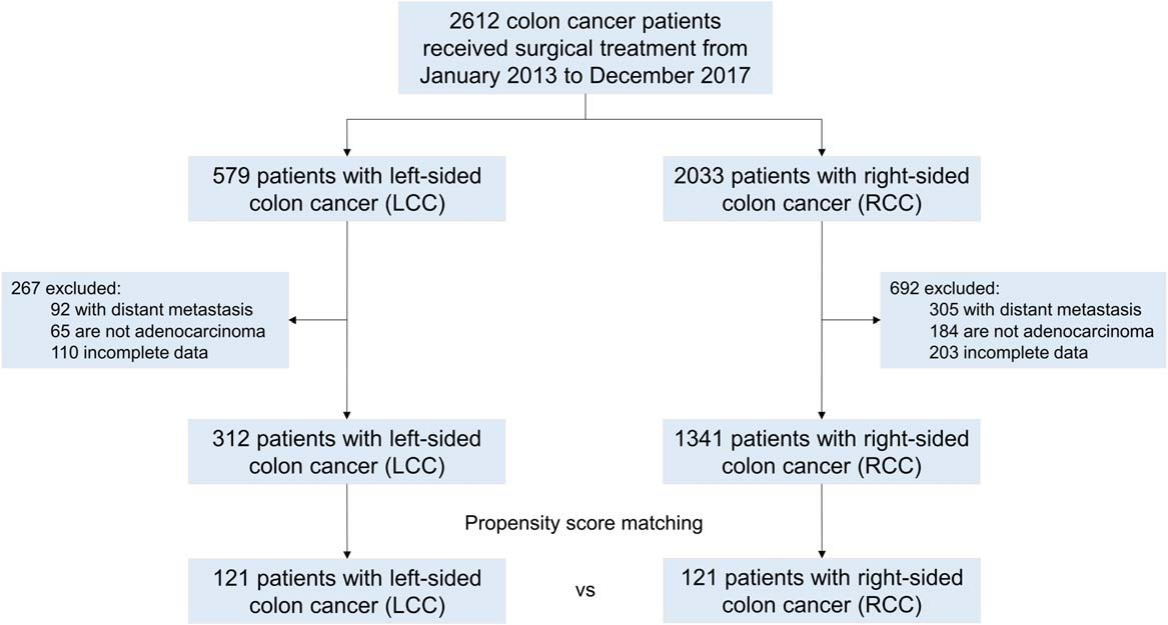
Flowchart of the study. This retrospective study involved 312 left-sided colon cancer and 1341 right-sided colon cancer patients following strict screening. After propensity score matching, 121 left-sided colon cancer and 121 right-sided colon cancer patients are finally involved. Patients who received neoadjuvant therapy before surgical research were excluded from the study.

The training set included 2612 patients who underwent surgical procedures at multiple medical centers from January 2013 to December 2017 were retrospectively analyzed. All patients underwent radical resection of colon cancer, and the process of inclusion and exclusion was shown in Figure 1. This study has been approved by the ethics committee of each medical center, and all patients signed preoperative surgical consent forms. Patients who met the following criteria were involved in this study: greater than or equal to 18 years old; patients underwent radical resection of colon cancer, and adenocarcinoma was confirmed by postoperative pathology; did not receive any other treatment before surgery; lymph node dissection number greater than or equal to 12; cooperate with follow-up. Patients with the following conditions were excluded: Distant metastasis (M1); Postoperative pathology proved nonadenocarcinoma; follow-up data were incomplete or missing. A total of 312 LCC and 1341 RCC patients following strict screening was selected. After propensity score matching, 121 LCC and 121 RCC patients were finally involved. Using the same criteria, the external validation set included 64 LCC patients and 64 RCC patients. This study was approved by the ethics committee of each medical center, and all patients signed preoperative surgical consent forms.

### Registration

According to the Helsinki Declaration, this study was registered at ResearchRegistry.com. This is a retrospective cohort study. This study was approved by the institutional research ethics committee of the corresponding center. All procedures performed in our study were in line with the strengthening the reporting of cohort, cross-sectional and case-control studies in surgery (STROCSS) criteria^23^, Supplemental Digital Content 1, http://links.lww.com/JS9/A630.

### Observation index

Basic information about the patient including age, sex, tumor size, and tumor site, etc, was collected. RCC refers to the cecum, ascending colon, and transverse colon near the liver. LCC refers to the transverse colon near the spleen, descending colon, and sigmoid colon. Postoperative pathological reports were collected, which included various pathological features such as tumor size, tumor differentiation (well/moderate/poor), DNA mismatch repair (dMMR/pMMR), T stage (T1/T2/T3/T4), N stage (N0/N1/N2), AJCC stage (all re-staged according to the eighth version of TNM stage, (N0/N1/N2), venous invasion (negative/positive), perineural invasion (negative/positive), and tumor budding (negative/weak/moderate/strong). All patients were routinely followed up for 5 years after surgery.

### Evaluation of TLS

Hematoxylin-eosin sections from the aforementioned patients were reviewed, and sections containing tumor and surrounding normal tissues were analyzed. The tumor samples were made into formalin-fixed, paraffin-embedded specimens. formalin-fixed, paraffin-embedded tissue blocks were sectioned at 4 µm for staining purposes. To further evaluate the cellular composition of TLS in LCC and RCC, immunohistochemical (IHC) staining was performed on the tissue sections according to the experimental methods described previously^21^. IHC staining was performed to identify helper T cells (Abcam, ab133616, rabbit monoclonal to CD4, 1:200), cytotoxic T cells (Abcam, ab178089, rabbit monoclonal to CD8 alpha, 1:100), regulatory T cells (Abcam, ab20034, mouse monoclonal to FOXP3, 1:500), memory T cells (Abcam, mouse monoclonal to CD45RO, 1:1,000), B cells (Abcam, ab78237, rabbit monoclonal to CD20, 1:100), dendritic cells (Abcam, ab52632, rabbit monoclonal to CD11c, 1:500), natural killer cells (Abcam, ab224703, rabbit monoclonal to NCR1, 1:1000), follicular dendritic cells (Abcam, ab75985, rabbit monoclonal to CD21, 1:100), macrophage (Wuhan Sevicebio Technology Co., Ltd, GB113150, rabbit monoclonal to CD68, 1:500), and tumor-associated neutrophil (Santa Cruz, SC-21702, mouse monoclonal to CD15, 1:100). Following the staining procedure, all sections were reviewed by two independent observers who assessed the number and location of TLS. These observers were fully blinded to the clinical characteristics of the patients. The tumor-related TLS were defined as those within 7 mm from the tumor border including the tumor region^11^. The area of tumor-related TLS was calculated by Image J. TLS density was calculated as the number of TLS per mm^2^ of tumor-related TLS region in the slide. The evaluation of TLS maturity was based on the method outlined in a previous study^13^. The numbers of peri-tumoral immune cells, which did not belong to the TLS, were estimated in five random high magnification fields (×200). The percentage of each cell component in a TLS was calculated as the number of cells of each type in all nucleated cells in the TLS. Then an average value of every cell component in TLS in the whole slide was used as the representative value of a patient.

### Statistical analyses

Statistical analyses were performed using R Studio (version 3.6.3) and SPSS (version 26.0). The χ^2^ or Fisher exact test was used to compare categorical variables and the *t*-test or analysis of variance was used to compare continuous variables. Receiver operating characteristic curves (ROC) were established to determine the cutoff values to discriminate patients with or without death. Kaplan–Meier curves of OS was plotted for TLS density. Variables were examined first by using the univariate Cox regression analysis, and significant variables were forced into the multivariate Cox regression analysis. The 1:1 propensity score matching was carried out by SPSS26.0 software, and the caliper value was 0.02. Both precise matching and fuzzy matching were included in the data after matching. A nomogram for predicting the OS was built using the R library ‘rms’ package. The nomogram was first internally validated using a bootstrap method and then externally validated in the independent cohorts.

## Results

### Comparison of clinicopathological characteristics of involved colon cancer patients between training cohort and validation cohort

We retrospectively analyzed 121 patients with LCC and 121 patients with RCC based on propensity score matching and these patients were used as an internal validation set. Sixty-four patients with LCC and 64 patients with RCC were used as an external validation set. There was no significant difference between the training and validation cohorts in demographic and clinical characteristics (Table 1).

**Table 1:** Comparison between training cohort and validation cohort of clinicopathological characteristics of involved colon cancer patients.

	LCC			RCC		
	Training cohort (*n*=121)	Validation cohort (*n*=64)	*P*	Training cohort (*n*=121)	Validation cohort (*n*=64)	*P*
Age (years), means±SD	61.16±12.369	60.422±11.759	0.696	61.61±13.291	60.969±12.786	0.752
Sex, *n* (%)			0.546			0.347
Male	81 (66.9%)	40 (62.4%)		91 (75.2%)	44 (68.8%)	
Female	40 (33.1%)	24 (37.6%)		30 (24.8%)	20 (31.2%)	
Tumor size, *n* (%)			0.845			0.421
<5 cm	83 (68.6%)	43 (67.2%)		68 (56.2%)	32 (50.0%)	
≥5 cm	38 (31.4%)	21 (32.8%)		53 (43.8%)	32 (50.0%)	
Tumor differentiation, *n* (%)			0.169			0.803
Well	24 (19.8%)	3 (4.6%)		12 (9.9%)	7 (11.0%)	
Moderate	63 (52.1%)	43 (67.2%)		85 (70.2%)	45 (70.2%)	
Poor	34 (28.1%)	18 (28.2%)		24 (19.8%)	12 (18.8%)	
DNA mismatch repair, *n* (%)			0.654			0.847
dMMR	16 (13.2%)	7 (10.9%)		25 (20.7%)	14 (21.9%)	
pMMR	105 (86.8%)	57 (89.1%)		96 (79.3%)	50 (78.1%)	
T stage, *n* (%)			0.972			0.559
T1	9 (7.4%)	8 (12.4%)		11 (9.1%)	7 (11.0%)	
T2	20 (16.5%)	10 (15.6%)		23 (19.0%)	15 (23.4%)	
T3	73 (60.3%)	32 (50.0%)		69 (57.0%)	32 (50.0%)	
T4	19 (15.7%)	14 (21.8%)		18 (14.9%)	10 (15.6%)	
N stage, n (%)			0.949			0.511
N0	37 (30.6%)	22 (34.4%)		55 (45.5%)	34 (53.1%)	
N1	52 (43.0%)	22 (34.4%)		41 (33.9%)	16 (25.0%)	
N2	32 (26.4%)	20 (31.3%)		25 (20.7%)	14 (21.9%)	
AJCC stage, *n* (%)			0.316			0.687
I	21 (17.4%)	10 (15.6%)		27 (22.3%)	14 (21.8%)	
II	31 (25.6%)	12 (18.8%)		45 (37.2%)	18 (28.2%)	
III	69 (57.0%)	42 (65.6%)		49 (40.5%)	32 (50.0%)	
Venous invasion, *n* (%)			0.569			0.936
Negative	88 (72.7%)	44 (68.8%)		92 (76.0%)	49 (76.6%)	
Positive	33 (27.3%)	20 (31.2%)		29 (24.0%)	15 (23.4%)	
Perineural Invasion, *n* (%)			0.228			0.729
Negative	79 (65.3%)	36 (56.2%)		75 (62.0%)	38 (59.4%)	
Positive	42 (34.7%)	28 (43.8%)		46 (38.0%)	26 (40.6%)	
Tumor budding, *n* (%)			0.466			0.920
Negative	23 (19.0%)	9 (14.1%)		15 (12.4%)	8 (12.5%)	
Weak	57 (47.1%)	31 (48.4%)		73 (60.3%)	38 (59.4%)	
Moderate	27 (22.3%)	16 (25.0%)		28 (23.1%)	15 (23.4%)	
Strong	14 (11.6%)	8 (12.5%)		5 (4.1%)	3 (4.7%)	

LCC Left-side colon cancer, RCC Right-side colon cancer, dMMR Deficient mismatch repair, pMMR Proficient mismatch repair.

### Comparison of clinicopathological characteristics between LCC and RCC

In the training cohort, univariate analysis was performed for LCC and RCC. Results indicated that there were no significant differences in age, sex, tumor differentiation, DNA mismatch repair, T stage, venous invasion, perineural invasion, and tumor budding (*P*>0.05). However, significant statistical differences were observed between the two groups in terms of tumor size (*P*=0.047), N stage (*P*=0.032), and AJCC stage (*P*=0.021) (Table 2). Similar results were found in the validation cohort. (Table S1, Supplemental Digital Content 7, http://links.lww.com/JS9/A645).

**Table 2:** Clinicopathological characteristics of involved colon cancer patients.

	LCC patients (*n*=121)	RCC patients (*n*=121)	*P*
Age (years), means±SD	61.16±12.369	61.61±13.291	0.783
Gender, *n* (%)			0.156
Male	81 (66.9%)	91 (75.2%)	
Female	40 (33.1%)	30 (24.8%)	
Tumor size, *n* (%)			**0.047** *
<5 cm	83 (68.6%)	68 (56.2%)	
≥5 cm	38 (31.4%)	53 (43.8%)	
Tumor differentiation, *n* (%)			0.938
Well	24 (19.8%)	12 (9.9%)	
Moderate	63 (52.1%)	85 (70.2%)	
Poor	34 (28.1%)	24 (19.8%)	
DNA mismatch repair, *n* (%)			0.123
dMMR	16 (13.2%)	25 (20.7%)	
pMMR	105 (86.8%)	96 (79.3%)	
T stage, *n* (%)			0.525
T1	9 (7.4%)	11 (9.1%)	
T2	20 (16.5%)	23 (19.0%)	
T3	73 (60.3%)	69 (57.0%)	
T4	19 (15.7%)	18 (14.9%)	
N stage, *n* (%)			**0.032** *
N0	37 (30.6%)	55 (45.5%)	
N1	52 (43.0%)	41 (33.9%)	
N2	32 (26.4%)	25 (20.7%)	
AJCC stage, *n* (%)			**0.021** *
I	21 (17.4%)	27 (22.3%)	
II	31 (25.6%)	45 (37.2%)	
III	69 (57.0%)	49 (40.5%)	
Venous invasion, *n* (%)			0.556
Negative	88 (72.7%)	92 (76.0%)	
Positive	33 (27.3%)	29 (24.0%)	
Perineural Invasion, *n* (%)			0.593
Negative	79 (65.3%)	75 (62.0%)	
Positive	42 (34.7%)	46 (38.0%)	
Tumor budding, *n* (%)			0.686
Negative	23 (19.0%)	15 (12.4%)	
Weak	57 (47.1%)	73 (60.3%)	
Moderate	27 (22.3%)	28 (23.1%)	
Strong	14 (11.6%)	5 (4.1%)	

*P<0.05.

### TLS features between LCC and RCC

**Figure 2:**
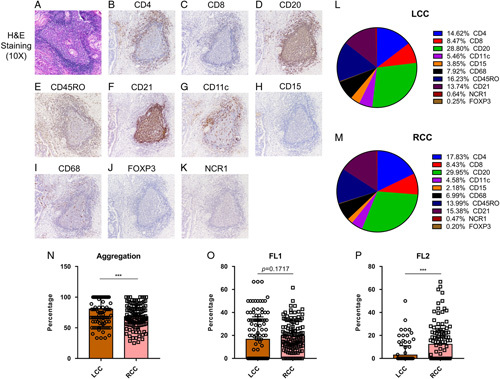
H&E sections showing TLS and different immune cells in TLS. (A) Representative figure of H&E slides to show TLS. (B-K) IHC results showed the different immune cells, which formed the TLS, including (B) CD4^+^ T cells, (C) CD8^+^ T cells, (D) CD20^+^ B cells, (E) CD45RO^+^ memory T cells, (F) CD21^+^ follicular dendritic cells, (G) CD11c^+^ dendritic cells, (H) CD15^+^ granulocytes, (I) CD68^+^ macrophages, (J) FOXP3^+^ Treg cells, and (K) NCR1^+^ natural killer cells. (L and M) Pie chart figure showed the distribution of various immune cells forming the TLS between LCC and RCC. (N–P) Scatter plots showed different TLS maturity status between LCC and RCC. LCC, left-sided colon cancer; RCC, right-sided colon cancer.

**Figure 3:**
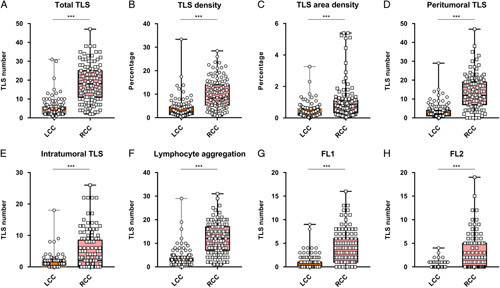
Scatter plot showed the numerous differentiations of TLS infiltration between left-sided colon cancer and right-sided colon cancer. LCC, left-sided colon cancer; RCC, right-sided colon cancer. ***P<0.001.

Hematoxylin-eosin staining was used to explore the number, maturity, density, and location of TLS in patients with LCC and RCC. Both peritumoral and intratumoral TLS were counted, but no TLS was found within the tumor parenchyma. TLS exhibited a variety of sizes and shapes: Aggregates (Agg) were typically squashed, elongated, or teardrop-shaped, while primary follicles (FL-1) were generally round or oval, and secondary follicles (FL-2) contained a germinal center (Fig. 2A). The results revealed that the number of TLS, Agg TLS, FL1 TLS, FL2 TLS, TLS density, TLS area density, intratumoral TLS, and peritumoral TLS in RCC were significantly higher than those observed in LCC. (Table 3 and Fig. 3). Similar results were obtained in the validation cohort (Table S2, Supplemental Digital Content 7, http://links.lww.com/JS9/A645). Analysis of TLS maturity indicated that the percentage of Agg in LCC was higher than that in RCC (Fig. 2N). The percentage of FL1 in LCC was identical to that in RCC (Fig. 2O), while the percentage of FL2 in LCC was lower than that in RCC (Fig. 2P). This indicated that TLS maturity in RCC was higher than that in LCC.

**Table 3:** TLS features of involved colon cancer patients.

	LCC patients (*n*=121)	RCC patients (*n*=121)	***P
TLS number, mean±SD	4.93±4.752	18.35±9.593	**<0.001**
Agg TLS number	3.74±3.698	12.12±6.441	**<0.001**
FL1 TLS number	0.97±1.460	3.83±3.420	**<0.001**
FL2 TLS number	0.22±0.612	2.40±3.581	**<0.001**
TLS density, mean±SD	3.62±4.112	10.08±6.076	**<0.001**
TLS area density (%), mean±SD	0.47±0.460	0.97±1.120	**<0.001**
Intratumoral TLS number, mean±SD	1.50±2.141	4.79±5.959	**<0.001**
Peritumoral TLS number, mean±SD	3.44±3.772	13.54±8.668	**<0.001**

***P<0.001.

Subgroup analysis suggested that TLS density in RCC was higher than that in LCC at T1, T2, and T3 stage, while there was no difference at T4 stage (Supplementary Fig 1A, Supplemental Digital Content 2, http://links.lww.com/JS9/A631). Intragroup analysis of both LCC and RCC showed that TLS density decreased gradually from T1 to T4 stage (Supplementary Fig 1B–C, Supplemental Digital Content 2, http://links.lww.com/JS9/A631). This suggested that different tumor sites (LCC/RCC) and different T stages may affect TLS density. Analysis of N stage subgroup showed that TLS density in RCC was higher than that in LCC in the same N stage (Supplementary Fig 1D, Supplemental Digital Content 2, http://links.lww.com/JS9/A631). However, intragroup analysis of LCC and RCC showed that there was no significant difference in TLS density across various N stages (*P*>0.05) (Supplementary Fig 1E–F, Supplemental Digital Content 2, http://links.lww.com/JS9/A631). Subgroup analysis of the same T stage in the LCC or RCC group also revealed no statistical difference in TLS density across various N stages (*P*>0.05) (Supplementary Fig 2, Supplemental Digital Content 3, http://links.lww.com/JS9/A632). These results suggested that the N stage may not affect TLS density.

The proportion of immune subsets in TLS between LCC and RCC were investigated by IHC staining. IHC results clearly showed immune subsets involved in TLS, including CD4^+^ T cells, CD8^+^ T cells, CD20^+^ B cells, CD45RO^+^ memory T cells, CD21^+^ follicular dendritic cells, CD11c^+^ dendritic cells, CD15^+^ granulocytes, CD68^+^ macrophages, FOXP3^+^ Treg cells, and NCR1^+^ natural killer cells (Fig. 2 B–K). A pie chart illustrated the distribution of various immune subsets in TLS between LCC and RCC (Fig. 2 L–M). The proportion of cell components in TLS showed that the proportion of CD4^+^ T cells of TLS in RCC was significantly higher than that in LCC, while the proportions of CD45RO^+^ memory T cells and CD15^+^ granulocytes were lower than those in LCC. There was no significant difference in the proportion of other cells between LCC and RCC (Supplementary Fig 3, Supplemental Digital Content 4, http://links.lww.com/JS9/A633). Intra-group analysis suggested there was no significant difference of tumor-infiltrated immune cells among T1-T4 stages between LCC and RCC (Supplementary Fig 4, Supplemental Digital Content 5, http://links.lww.com/JS9/A634 and Supplementary Fig 5, Supplemental Digital Content 6, http://links.lww.com/JS9/A635).

### TLS density and TILs located at tumor margin between LCC and RCC

**Figure 4:**
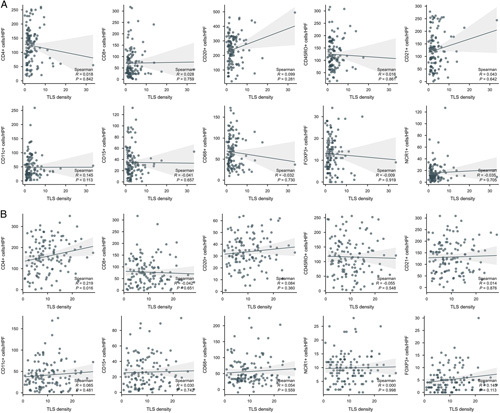
Scatter plots indicated the relationship between TLS density and various immune cells in (A) LCC and (B) RCC. Increasing TLS density was correlated to higher infiltration of CD4^+^ T cells in RCC, while the other plots did not show the association between TLS density and other tumor-infiltrating immune cells. LCC, left-sided colon cancer; RCC, right-sided colon cancer.

Next, we explored the relationship between the density of TLS in LCC/RCC and TILs located at the tumor margin. Results showed that there was no significant correlation between the TLS density and TILs in LCC (Fig. 4A). While increasing TLS density was correlated to higher infiltration of CD4^+^ T cells in RCC, other TILs subsets did not show the association with TLS density (Fig. 4B). Altogether, these results demonstrated that TLS in RCC may promote the antitumor immune effect in the tumor immune microenvironment.

### Association of TLS density with the survival of patients

**Figure 5:**
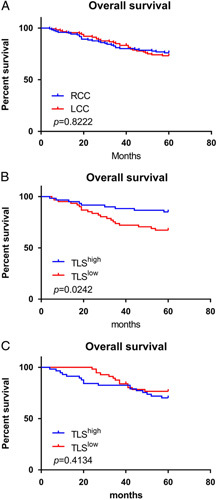
Kaplan–Meier plots showed the different prognosis between (A RCC and LCC patients; (B TLS^high^ and TLS^low^ patients in RCC group; and (C TLS^high^ and TLS^low^ patients in LCC group. In RCC group, patients with more TLS in tumor microenvironment had a better prognosis than TLS ^low^ patients. LCC, left-sided colon cancer; RCC, right-sided colon cancer.

To better clarify the effects TLS exerting in the tumor immune microenvironment, 5-year OS of patients was recorded. Results showed that there was no significant difference in 5-year OS between RCC and LCC (Fig. 5A). The TLS^High^ and TLS^Low^ groups were established based on the average number of TLS present in the samples. In RCC group, univariate analysis suggested that TLS^High^ group had a lower T stage and AJCC stage, a lower positive lymph node rate, and a lower positive vascular invasion rate (Table 4), and patients with a higher number of TLS in tumor microenvironment had a better prognosis than TLS^low^ patients (Fig. 5B). Univariate analysis suggested that the TLS^High^ group was younger and had a lower AJCC stage (Table 5), while TLS density had no significance correlation with OS (Fig. 5C).

**Table 4:** Clinicopathological characteristics between TLS^high^ and TLS^low^ group of RCC patients.

	TLS^high^ patients (*n*=60)	TLS^low^ patients (*n*=61)	*P*
Age (year), means±SD	59.83±12.717	63.36±13.711	0.145
Sex, *n* (%)			0.712
Male	46 (76.7%)	45 (73.8%)	
Female	14 (23.3%)	16 (26.2%)	
Tumor size, *n* (%)			0.117
<5 cm	38 (63.3%)	30 (49.2%)	
≥5 cm	22 (36.7%)	31 (50.8%)	
Differentiation, *n* (%)			0.203
Well	9 (15.0%)	3 (4.9%)	
Moderate	40 (66.7%)	45 (73.8%)	
Poor	11 (18.3%)	13 (21.3%)	
DNA mismatch repair, *n* (%)			0.472
dMMR	14 (23.3%)	11 (18.0%)	
pMMR	46 (76.7%)	50 (82.0%)	
T stage, *n* (%)			0.001**
T1	9 (15.0%)	2 (3.3%)	
T2	11 (18.3%)	12 (19.7%)	
T3	40 (66.7%)	29 (47.5%)	
T4	0 (0.0%)	18 (29.5%)	
N stage, *n* (%)			0.011*
N0	33 (55%)	22 (36.1%)	
N1	20 (33.3%)	21 (34.4%)	
N2	7 (11.7%)	18 (29.5%)	
AJCC stage, *n* (%)			0.002**
I	19 (31.7%)	8 (13.1%)	
II	24 (40.0%)	21 (34.4%)	
III	17 (28.3%)	32 (52.5%)	
Venous invasion, *n* (%)			0.022*
Negative	51 (85.0%)	41 (67.2%)	
Positive	9 (15.0%)	20 (32.8%)	
Perineural Invasion, *n* (%)			0.154
Negative	41 (68.3%)	34 (55.7%)	
Positive	19 (31.7%)	27 (44.3%)	
Tumor budding, *n* (%)			0.873
Negative	9 (15.0%)	6 (9.8%)	
Weak	33 (55.0%)	40 (65.6%)	
Moderate	18 (30.0%)	10 (16.4%)	
Strong	0 (0.0%)	5 (8.2%)	

*P<0.05.

**P<0.01.

**Table 5:** Clinicopathological characteristics between TLS^high^ and TLS^low^ group of LCC patients.

	TLS^high^ patients (*n*=61)	TLS^low^ patients (*n*=60)	*P*
Age (years), means±SD	58.48±12.679	63.88±11.520	0.016*
Sex, *n* (%)			0.747
Male	40 (65.6%)	41 (68.3%)	
Female	21 (34.4%)	19 (31.7%)	
Tumor size, *n* (%)			0.216
<5cm	45 (73.8%)	38 (63.3%)	
≥5cm	16 (26.2%)	22 (36.7%)	
Differentiation, *n* (%)			0.113
Well	21 (34.4%)	13 (21.7%)	
Moderate	30 (49.2%)	33 (55.0%)	
Poor	10 (16.4%)	14 (23.3%)	
DNA mismatch repair, *n* (%)			0.972
dMMR	8 (13.1%)	8 (13.3%)	
pMMR	53 (86.9%)	52 (86.7%)	
T stage, *n* (%)			0.112
T1	7 (11.5%)	2 (3.3%)	
T2	18 (29.5%)	2 (3.3%)	
T3	21 (34.4%)	52 (86.7%)	
T4	15 (24.6%)	4 (6.7%)	
N stage, *n* (%)			0.120
N0	22 (36.1%)	15 (25.0%)	
N1	26 (42.6%)	26 (43.3%)	
N2	13 (21.3%)	19 (31.7%)	
AJCC stage, *n* (%)			<0.001***
I	20 (32.8%)	1 (1.7%)	
II	14 (23.0%)	17 (28.3%)	
III	27 (44.3%)	42 (70.0%)	
Venous invasion, *n* (%)			0.170
Negative	41 (67.2%)	47 (78.3%)	
Positive	19 (32.8%)	13 (21.7%)	
Perineural Invasion, *n* (%)			0.752
Negative	39 (63.9%)	40 (66.7%)	
Positive	22 (36.1%)	20 (33.3%)	
Tumor budding, *n* (%)			0.487
Negative	15 (24.6%)	8 (13.3%)	
Weak	22 (36.1%)	35 (58.3%)	
Moderate	11 (18.0%)	16 (26.7%)	
Strong	13 (21.3%)	1 (1.7%)	

*P<0.05.

***P<0.001.

### univariate and multivariate analyses of risk factors associated with overall survival

In the training cohort, univariate analyses by Kaplan–Meier curves and log-rank tests showed that AJCC stage, differentiation tumor budding, venous invasion, perineural invasion, and TLS density were associated with the overall survival of patients with RCC. Multivariate analysis was then conducted and demonstrated that only AJCC stage, venous invasion, and TLS density were independent risk factors for the overall survival of patients with RCC (Table 6). Meanwhile, univariate analysis also showed that tumor size, AJCC stage, differentiation, tumor budding, venous invasion, and perineural invasion were associated with the overall survival of patients with LCC. Multivariate analysis was then performed and indicated that AJCC stage, differentiation, and tumor budding were independent risk factors for overall survival in patients with RCC (Table 7).

**Table 6:** Univariate and multivariate analysis for OS using cox regression in RCC.

		Univariate analysis		Multivariate analysis	
Characteristics	Total (*N*)	Hazard ratio (95% CI)	*P*	Hazard ratio (95% CI)	*P*
Age	121	1.019 (0.996–1.043)	0.105		
Sex	121		0.299		
Male	91	Reference			
Female	30	0.687 (0.330–1.430)	0.316		
Tumor size	121		0.328		
≤5 cm	68	Reference			
>5 cm	53	1.345 (0.744–2.431)	0.327		
AJCC stage	121		<0.001***		
Stage1	27	Reference		Reference	
Stage2	45	6.512 (1.510–28.082)	0.012*	5.688 (1.197–27.037)	0.029*
Stage3	49	9.122 (2.154–38.637)	0.003**	5.609 (1.227–25.649)	0.026*
Differentiation	121		0.018*		
Well	12	Reference		Reference	
Moderate	85	5.253 (0.716–38.535)	0.103	1.782 (0.229–13.852)	0.581
Poor	24	8.769 (1.146–67.082)	0.036*	2.534 (0.293–21.947)	0.399
Tumor budding	121		0.011*		
Negative	15	Reference		Reference	
Weak	73	3.740 (0.892–15.682)	0.071	2.592 (0.605–11.107)	0.199
Moderate	28	2.862 (0.618–13.250)	0.179	1.320 (0.263–6.625)	0.736
Strong	5	15.819 (2.871–87.172)	0.002**	4.199 (0.700–25.188)	0.116
Venous invasion	121		<0.001***		
Negative	92	Reference		Reference	
Positive	29	3.143 (1.719–5.746)	<0.001***	2.246 (1.144–4.407)	0.019*
Perineural invasion	121		0.004**		
Negative	75	Reference		Reference	
Positive	46	2.372 (1.309–4.297)	0.004**	1.095 (0.543–2.208)	0.801
MMR status	121		0.694		
dMMR	25	Reference			
pMMR	96	1.164 (0.541–2.503)	0.698		
TLS density	121	0.879 (0.824–0.939)	<0.001***	0.915 (0.852–0.982)	0.014*

*P<0.05.

**P<0.01.

***P<0.001.

**Table 7:** Univariate and multivariate analysis for OS using cox regression in LCC patients.

		Univariate analysis		Multivariate analysis	
Characteristics	Total (*N*)	Hazard ratio (95% CI)	*P*	Hazard ratio (95% CI)	*P*
Age	121	0.996 (0.974–1.019)	0.755		
Sex	121		0.364		
Male	81	Reference			
Female	40	1.288 (0.75–2.206)	0.358		
Tumor size	121		0.019*		
≤5 cm	83	Reference		Reference	
>5 cm	38	1.909 (1.127–3.232)	0.016*	1.638 (0.910–2.950)	0.100
AJCC stage	121		<0.001***		
Stage1	21	Reference		Reference	
Stage2	31	9.999 (1.300–76.929)	0.027*	10.674 (0.851–133.920)	0.067
Stage3	69	21.423 (2.948–155.691)	0.002**	20.853 (1.488–292.290)	0.024*
Differentiation	121		<0.001***		
Well	24	Reference		Reference	
Moderate	63	6.392 (1.519–26.894)	0.011*	5.500 (1.242–24.351)	0.025*
Poor	34	20.511 (4.864–86.499)	< 0.001***	14.221 (2.789–72.520)	0.001**
Tumor budding	121		< 0.001***		
Negative	23	Reference		Reference	
Weak	57	2.372 (0.807–6.974)	0.117	2.913 (0.911–9.318)	0.072
Moderate	27	6.686 (2.276–19.638)	<0.001***	3.287 (1.058–10.215)	0.040*
Strong	14	24.895 (7.795–79.510)	<0.001***	3.942 (1.016–15.299)	0.047*
Venous Invasion	121		<0.001***		
Negative	88	Reference		Reference	
Positive	33	4.611 (2.711–7.842)	<0.001***	1.652 (0.825–3.307)	0.157
Perineural invasion	121		<0.001***		
Negative	79	Reference		Reference	
Positive	42	4.494 (2.617–7.717)	<0.001***	1.855 (0.946–3.636)	0.072
MMR status	121		0.372		
dMMR	16	Reference			
pMMR	105	0.714 (0.350–1.455)	0.353		
TLS density	121	0.925 (0.844–1.015)	0.099	1.096 (0.943–1.273)	0.232

*P<0.05.

**P<0.01.

***P<0.001.

### Construction and validation of the nomogram

**Figure 6:**
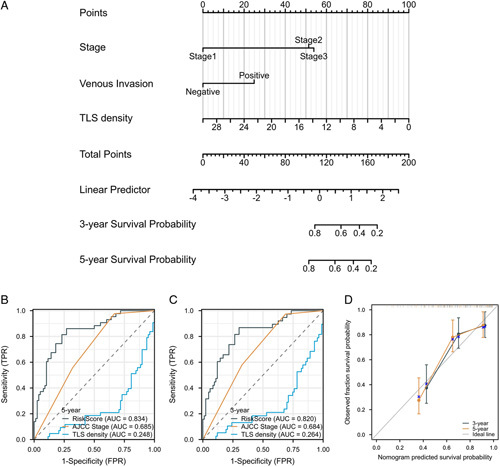
Construction and validation of the nomogram for RCC. (A) The nomogram to predict 3-year and 5-year survival probability for patients with RCC. (B and C) The ROC curve of 3- and 5-year survival probability in RCC patients, comparing the risk-score model with AJCC stage and TLS density alone. (D) The calibration curves of 3- and 5-year survival probability in RCC patients. RCC, right-sided colon cancer.

According to the results from the multivariate analysis, a nomogram clinical prediction model was constructed. Each independent risk factor was scored individually. Each individual score was added up to get the total score, and the probability corresponding to the total score was the probability of the model predicting the OS. As AJCC stage, venous invasion, and TLS density were identified as independent risk factors for overall survival in patients with RCC, these factors were integrated to develop the nomogram for RCC (Fig. 6A). By drawing the ROC curve, the predictive ability of the nomogram model was evaluated. The ROC curve of 3- and 5-year survival probability in RCC patients (AUC =0.820, AUC =0.834) displayed better results compared to the risk-score model with AJCC stage and TLS density alone (Fig. 6B–C). Besides, the calibration curve showed that the predicted results of the nomogram model had good consistency with the actual results (Fig. 6D).

**Figure 7:**
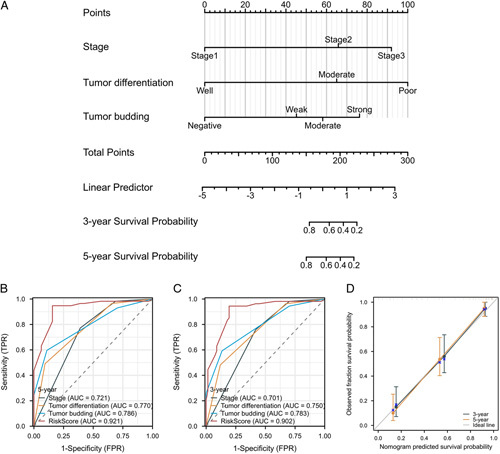
Construction and validation of the nomogram for LCC. (A) The nomogram to predict 3-year and 5-year survival probability for patients with LCC. (B and C) The ROC curve of 3- and 5-year survival probability in LCC patients, comparing the risk-score model with AJCC stage, tumor differentiation and tumor budding alone. (D) The calibration curves of 3- and 5-year survival probability in LCC patients. LCC, left-sided colon cancer.

Meanwhile, the construction and validation of the nomogram for LCC were also generated (Fig. 7A). Different from the results in the RCC group, the ROC curve of 3- and 5-year survival probability in LCC patients (AUC =0.902, AUC =0.921) were generated based on AJCC stage, tumor differentiation, and tumor budding (Fig. 7B–C). Besides, the prediction efficacy of calibration curves of 3- and 5-year survival in LCC patients was better than those generated based on AJCC stage, tumor differentiation, and tumor budding alone (Fig. 7D).

## Discussion

According to the physiological and anatomical characteristics of the colon, the colon can be divided into the left-sided colon and the right-sided colon, which are considered as two distinct organs, especially in the era of personalized medicine^24,25^. Within these categories, the clinical heterogeneity of LCC and RCC (such as metastasis, recurrence, prognosis, and treatment sensitivity) has been studied more specifically^26-28^. Patients with LCC benefit more from chemotherapy and targeted therapy and also experienced a better prognosis, while patients with RCC showed more promising results in immunotherapy^29^. Mirón Fernández *et al.* ^30^ pointed out that there were significant differences between RCC and LCC in terms of evolution, progression, complications, and survival. Compared with LCC, RCC had a more advanced N stage, a larger tumor volume, lower tumor differentiation, and a higher positive rate of lymphatic vascular invasion. However, detailed information about the immune index has not been fully addressed.

As an immune barrier around tumors, TLS has attracted increasing attention, and relevant meta-analysis indicated that higher expression of TLS in solid tumors was associated with longer OS and RFS, and a lower risk of tumor recurrence. Meanwhile, higher TLS expression was also associated with smaller tumor size, higher TILs infiltration, a lower tumor grade, and N stage^31^. We analyzed LCC and RCC data from internal and external validation sets to explore the correlation between TLS number, maturity, density, location, cell composition ratio, and prognosis. Firstly, we found that TLS density was significantly higher in RCC than that in LCC. Further intragroup analysis suggested that TLS density was correlated with the prognosis of patients with RCC but not for those with LCC. Di Caro *et al.* ^18^ showed that TLS density was significantly correlated with the prognosis of stage lll colorectal cancer patients, but not with stage ll colorectal cancer patients. In addition, a large number of studies have shown that higher TLS density was closely related to a better prognosis of colorectal cancer patients^12, 13,32^. Our study measured the density of TLS within 7 mm around the tumor^21^, and discovered for the first time the differences between TLS and prognosis in patients with LCC and RCC. Results showed that higher TLS density was associated with a good prognosis only in RCC patients, but not in LCC. This may be related to the bias of the exclusion criteria in which colorectal cancer and distant metastatic colon cancer patients were not involved in this study, suggesting that TLS density was more important in the prognostic process in patients with RCC. Subgroup analysis suggested that TLS density might be related to tumor location (LCC or RCC) and T stage, but not N stage. Therefore, these results suggested that TLS density may facilitate better immune responses in RCC, and tumor progression status were the main factors influencing TLS formation. Interestingly, the proportion of Agg in LCC was higher than that in RCC, while the proportion of FL-2 in RCC was higher than LCC, suggesting that TLS maturity may be correlated with tumor location.

Studies suggested that there were different immune surveillance modes for patients with LCC and RCC^33^. Tumor-associated immune microenvironment (TAIM) plays an important role in tumor development and immunotherapy response^34^, and the density of T lymphocyte infiltration in colon cancer is a reliable indicator to judge the risk of tumor recurrence and prognosis^35,36^. However, TAIM contains a wide range of cellular components and T lymphocyte identification is not an effective representative of the complex tumor immune environment. Studies on a variety of cancers have shown that inhibitory TAIM characterized by a series of immune cell and stromal cell infiltration had an important effect on tumor proliferation, metastasis, recurrence, and immunotherapy resistance^37^. To date, the immunocellular infiltration characteristics in LCC and RCC are unclear. We analyzed these aspects in LCC and RCC patients. As expected, TLS components were mainly CD20^+^ B cells, CD45RO^+^ memory T cells, CD4^+^ T cells, CD8^+^ T cells, NCR1^+^ NK cells, and CD11c^+^ DCs. We also identified immunosuppressive subsets such as CD15^+^ TANs and FOXP3^+^ Tregs. Subgroup analysis suggested that cell composition in TLS was not correlated with T stage, which was different from the results obtained for TLS density. We further explored the correlation between TLS density and the number of infiltrating lymphocytes at the tumor invasion edge. In RCC, the TLS density was significantly correlated with CD4^+^ T cells, and no significant correlation existed between the TLS density and TIL in LCC. Taken together, all these results suggested that the existence of TLS may exert anti-tumor immune responses partly through promoting the infiltration of CD4^+^ T cells.

Based on the above results, we included TLS density for multivariate analysis, and identified that TLS density, AJCC stage, and venous invasion were independent risk factors for the prognosis of patients with RCC. Subsequently, we developed and validated personalized nomograms incorporating TLS density, AJCC stage, and venous invasion to predict the OS probability for patients with RCC, which displayed a superior efficacy comparing with current available prediction models. Considering that TLS was not correlated with the prognosis of nonmetastatic LCC patients, we further explored the independent risk factors for the prognosis of these patients, with tumor budding, AJCC stage, and tumor differentiation status being independent risk factors identified. A nomogram based on the above factors to predict the prognosis of patients with LCC was generated and accurately predict the 3-year and 5-year OS. As for immune response, currently recognized DNA mismatch repair related conditions play an important role in the prognosis of patients with CRC and can be used as one of the molecules to guide treatment and predict prognosis^38,39^. No relevant studies involving large volume patient with LCC and RCC have been reported. Our results identified a higher proportion of dMMR status in patients with RCC than that with LCC (20.7 vs. 13.2%), while there was no significant correlation between MMR status and TLS density. Posch *et al.* ^13^ showed that although mismatch repair genes were associated with the formation of TLS in stage II and III colorectal cancer, TLS density was not correlated with tumor grade, stage, and age. The explanation for results from ours and others may be related to different inclusion criteria.

## Conclusion

The characteristics of TLS were different in patients with LCC and RCC, with TLS density being an independent risk factor only in RCC patients. A nomogram incorporating the TLS density, the AJCC eighth TNM stage, and venous invasion could more quickly and accurately predict the probability of OS at 3- and 5-year in RCC patients. With regard to LCC patients, tumor budding, the AJCC eighth TNM stage, and differentiation were identified as independent risk factors, and a nomogram based on these factors displayed superior prediction efficacy in the training and external validation set. Therefore, in addition to previously reported aspects, immune status involving TLS characteristics in patients with RCC and LCC were also different and future prediction and development of treatment strategies should take these into consideration.

## Ethical approval

This study has been approved by the ethics committee of Nanjing Drum Tower Hospital (2019-307-01), Sir Run Run Hospital of Nanjing Medical University (2019- SR-017), Geriatric Hospital of Nanjing Medical University (2019YFC2002000), and the Second Affiliated Hospital of Nanjing Medical University (2019-KY-173). In accordance with the ethics committee’s regulations, informed consent was obtained from patients included in this study.

## Sources of funding

This study was supported by grants from the National Nature Science Foundation of China (81970500, XF Shen), and the fundings for Clinical Trials from the Affiliated Drum Tower Hospital, Medical School of Nanjing University (2022- LCYJ-PY-33, YH Mao).

## Author contribution

X.S., C.Y., and Q.L.: designed the study concept; Y.M.: analyzed and interpreted the data, and wrote the final version of article; Y.M., X.W., L.X., and M.D: collected the original data and performed manuscript editing; P.S., J.M., C.L., and S.S.: edited the figure legends and analyzed follow-up of patients. All authors contributed to study design critically reviewed the first draft, approved the final version and agreed to be accountable for the work.

## Conflicts of interest disclosure

The authors declare that there are no conflict of interests existing.

## Research registration unique identifying number (UIN)

1. Name of the registry: Prediction values of Tertiary Lymphoid Structures in the prognosis of patients with left- and right-sided colon cancer: A Multicenter Propensity Score-Matched Study.
2. Unique Identifying number or registration ID: researchregistry8812.
3. Hyperlink to your specific registration (must be publicly accessible and will be checked): https://www.researchregistry.com/browse-theregistry#home/registrationdetails/642be0efdce8470028749b56/.

## Guarantor

Xiaofei Shen, Chunzhao Yu and Qiang Li.

## Data availability statement

All data included in this study are available upon request by contact with the corresponding author.

## Provenance and peer review

Not commissioned, externally peer reviewed.

## Acknowledgements

The authors would like to thank all the staff and patients in this study.

## Supplementary Material
