# Supplementary figures and images for: Prediction values of tertiary lymphoid structures in the prognosis of patients with left- and right-sided colon cancer: a multicenter propensity score-matched study
Source: Int J Surg. 2023 May 26;109(8):2344–58. doi: 10.1097/JS9.0000000000000483 (PMC10442147; doi:10.1097/JS9.0000000000000483)

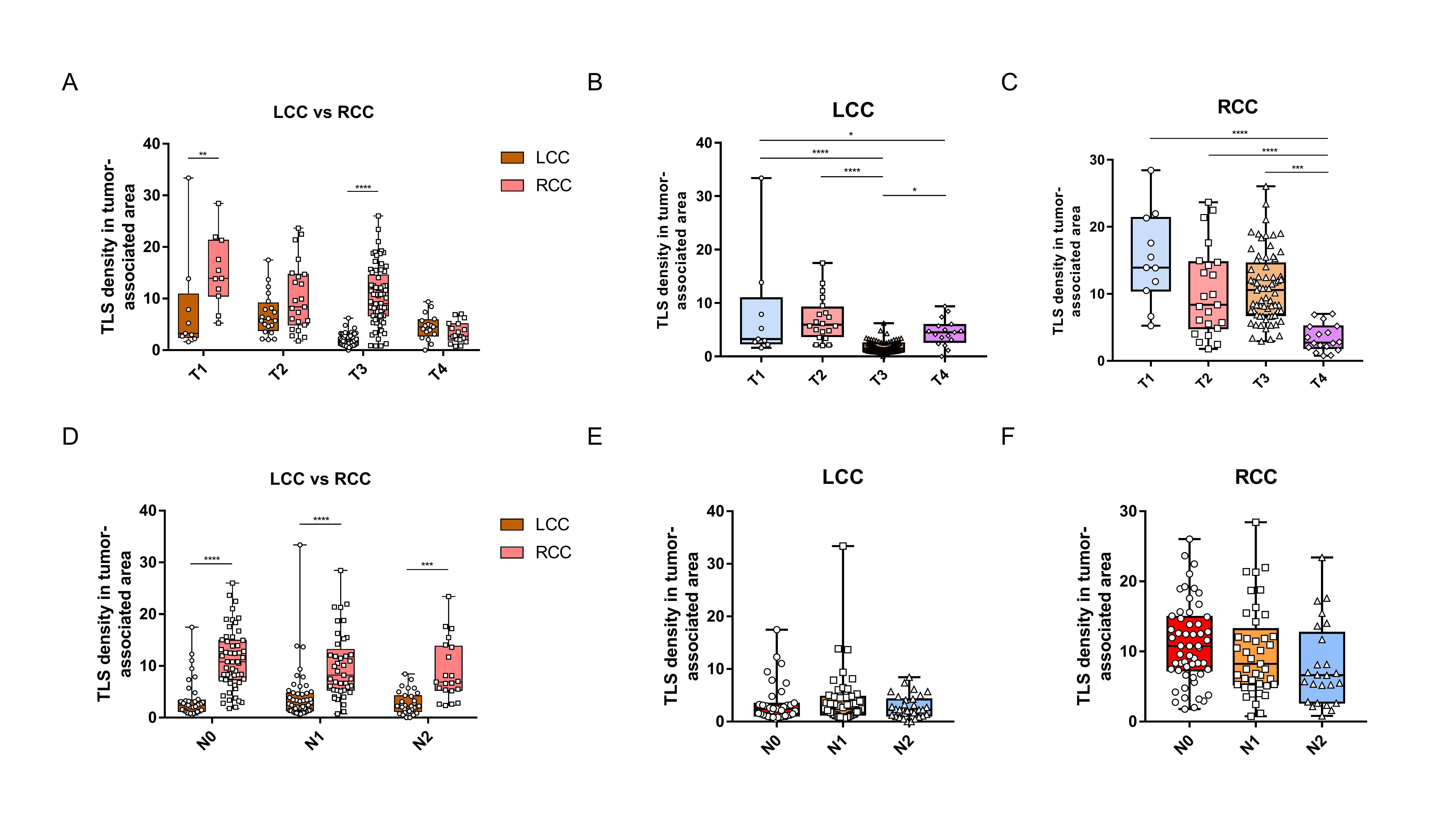

Supplement: Supplementary file 2 [file js9-109-2344-s002.jpg]

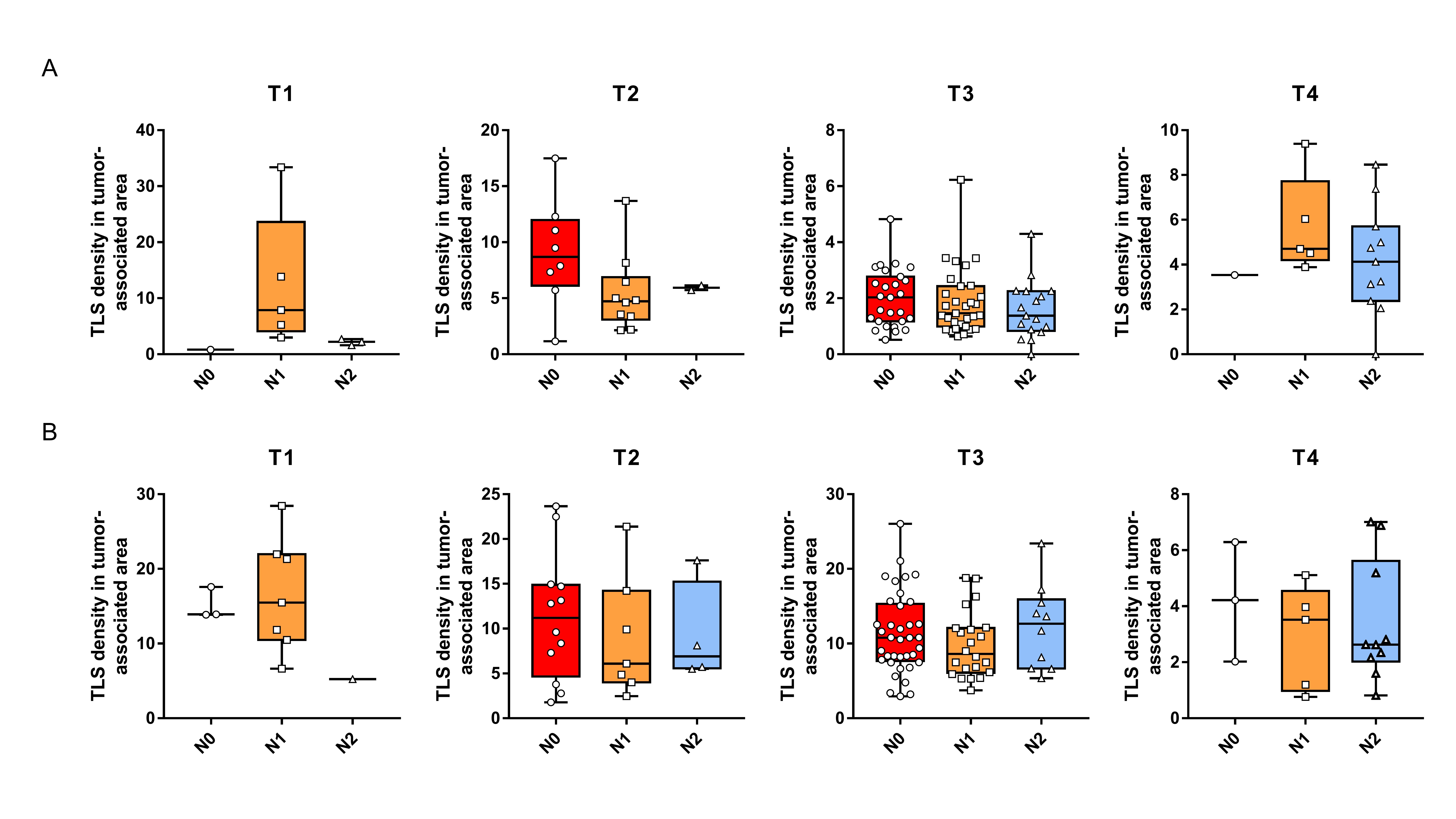

Supplement: Supplementary file 3 [file js9-109-2344-s003.jpg]

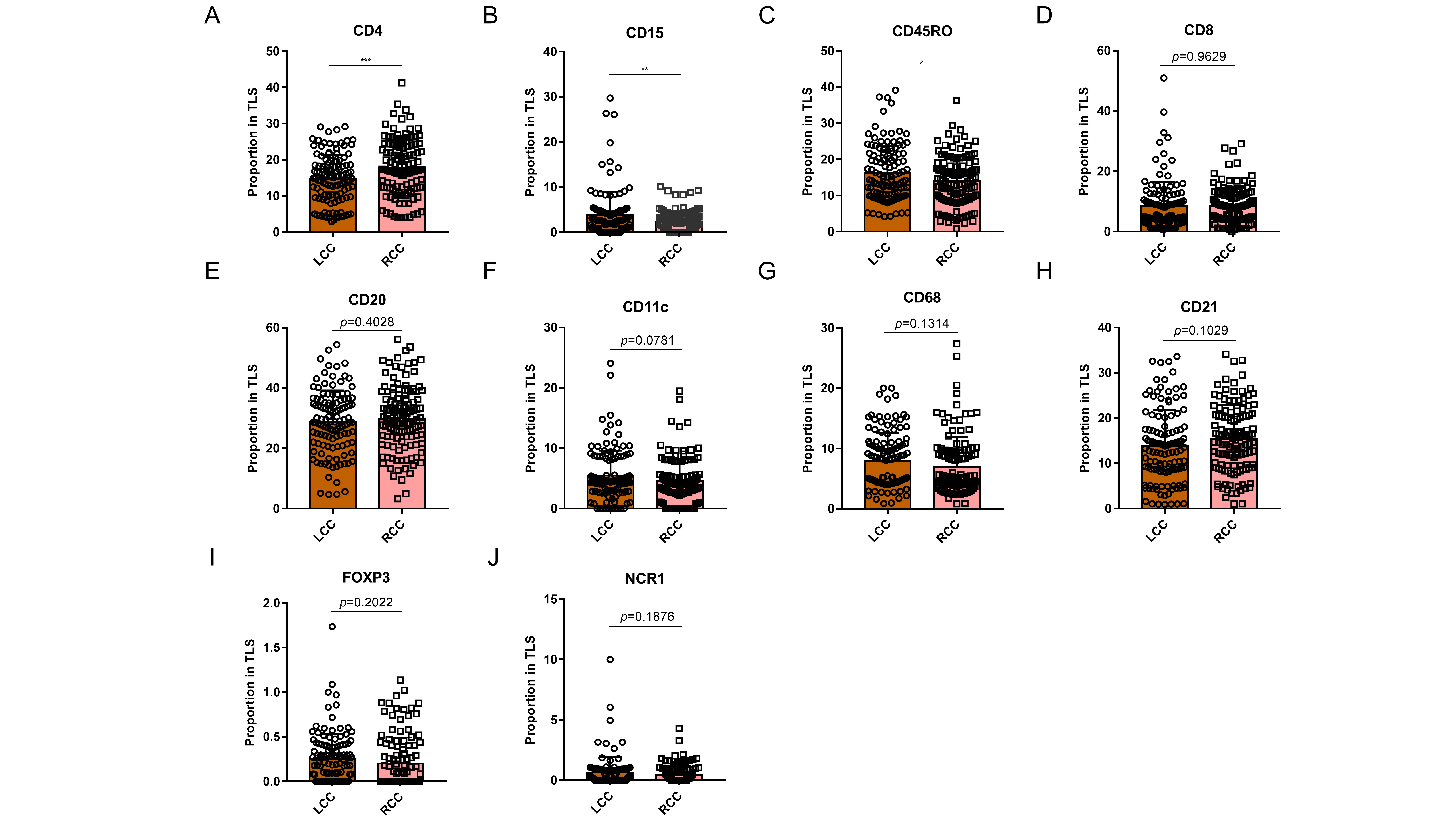

Supplement: Supplementary file 4 [file js9-109-2344-s004.jpg]

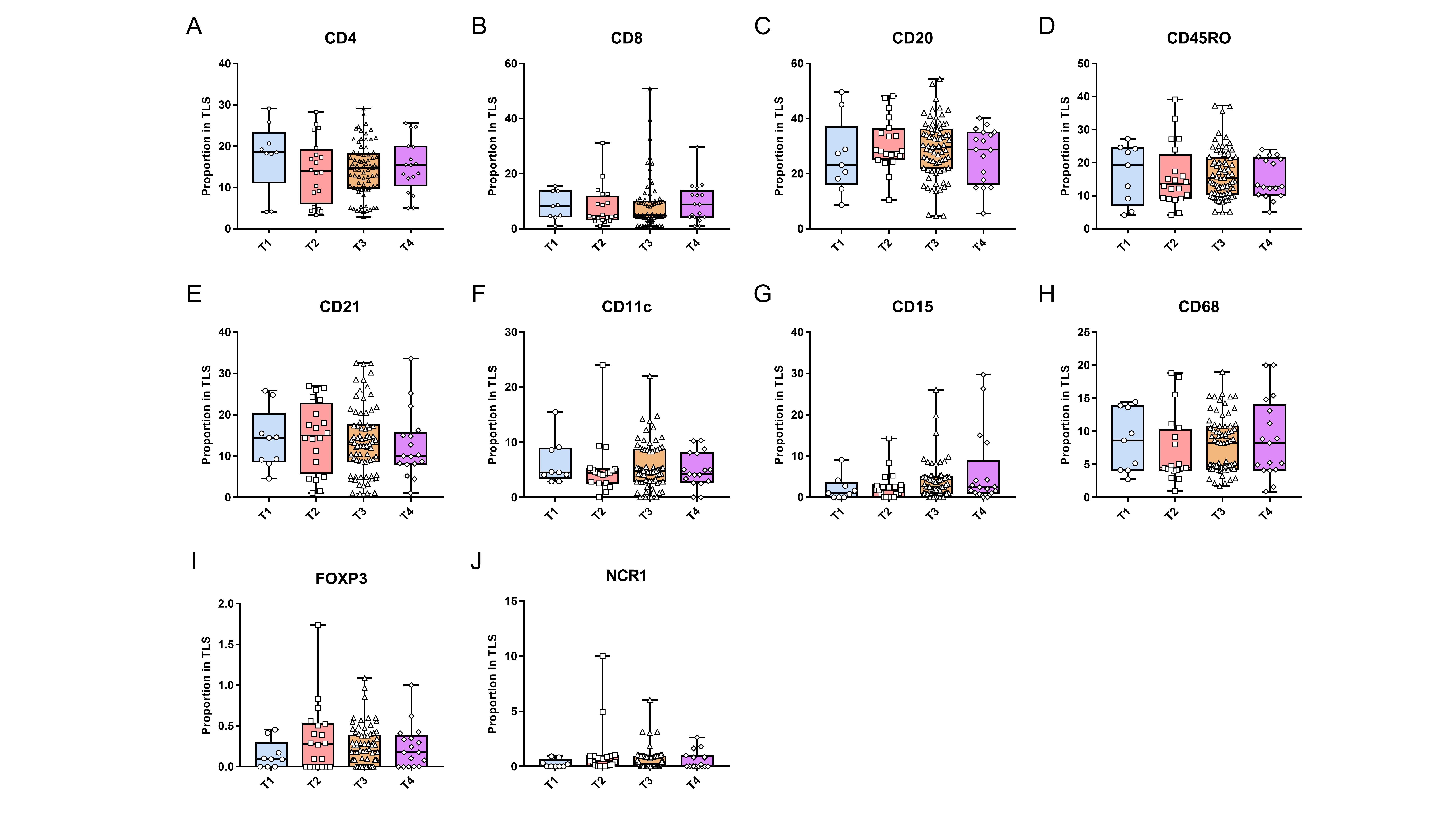

Supplement: Supplementary file 5 [file js9-109-2344-s005.jpg]

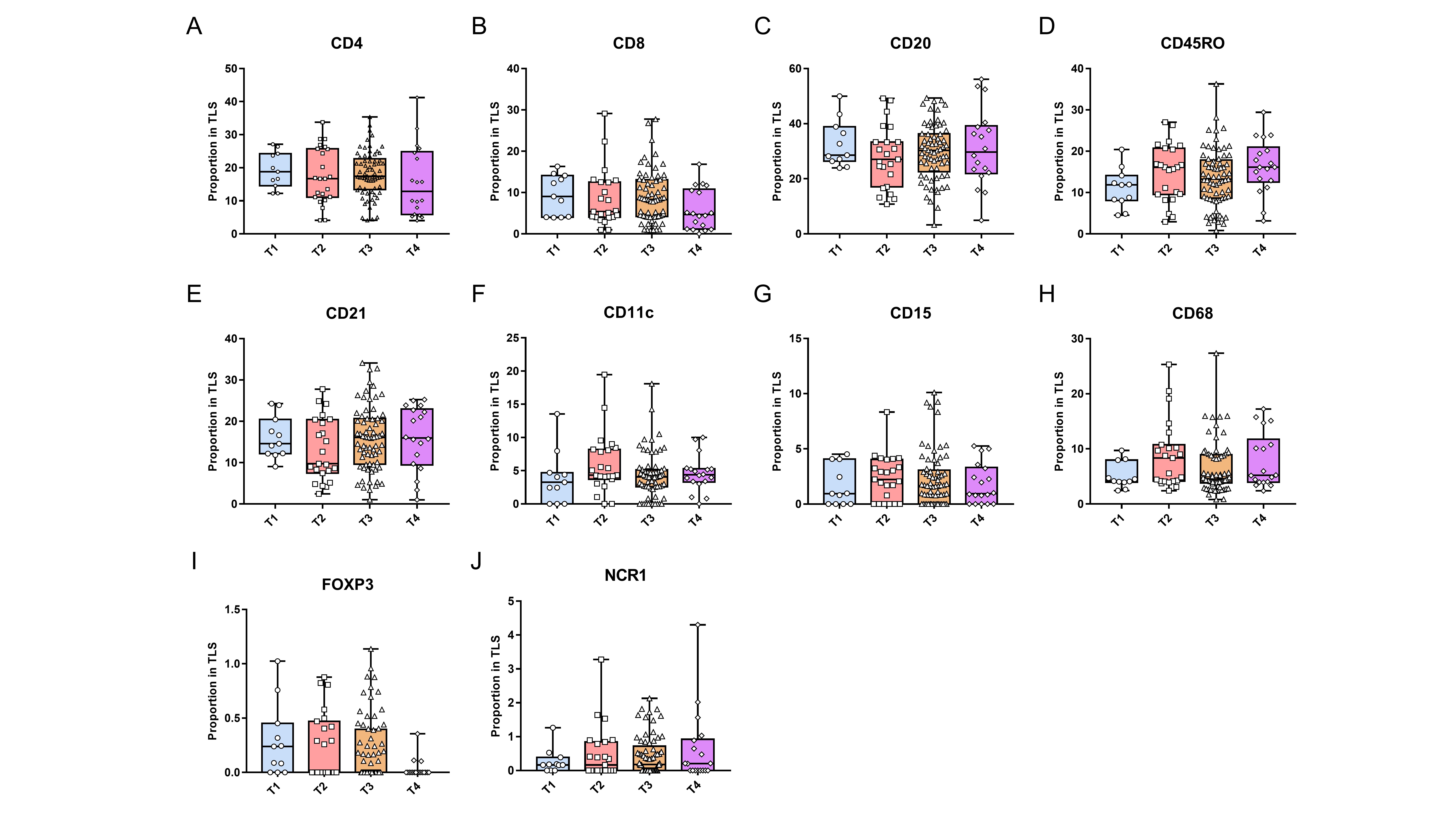

Supplement: Supplementary file 6 [file js9-109-2344-s006.jpg]
