# Supplementary material for: Prediction values of tertiary lymphoid structures in the prognosis of patients with left- and right-sided colon cancer: a multicenter propensity score-matched study
Source: Int J Surg. 2023 May 26;109(8):2344–58. doi: 10.1097/JS9.0000000000000483 (PMC10442147; doi:10.1097/JS9.0000000000000483)
Supplement: Supplementary file 7 [file js9-109-2344-s007.docx]

Table S1. Clinicopathological characteristics of involved colon cancer patients in validation cohort.

| Characteristics | LCC patients  (n=64) | RCC patients  (n=64) | P value |
| --- | --- | --- | --- |
| Age(years), mean ± SD | 60.422±11.759 | 60.969±12.786 | 0.802 |
| Gender, n (%) |  |  | 0.457 |
| male | 40(62.4%) | 44(68.8%) |  |
| female | 24(37.6%) | 20(31.2%) |  |
| **Tumor size, n (%)** |  |  | **0.048^*^** |
| <5cm | 43(67.2%) | 32(50.0%) |  |
| ≥5cm | 21(32.8%) | 32(50.0%) |  |
| Tumor differentiation, n (%) |  |  | 0.107 |
| Well | 3(4.6%) | 7(11.0%) |  |
| Moderate | 43(67.2%) | 45(70.2%) |  |
| Poor | 18(28.2%) | 12(18.8%) |  |
| DNA mismatch repair, n (%) |  |  | 0.095 |
| dMMR | 7(5.5%) | 14(10.9%) |  |
| pMMR | 57(44.5%) | 50(39.1%) |  |
| T stage, n (%) |  |  | 0.217 |
| T1 | 8(12.4%) | 7(11.0%) |  |
| T2 | 10(15.6%) | 15(13.4%) |  |
| T3 | 32(50%) | 32(50%) |  |
| T4 | 14(21.8%) | 10(15.6%) |  |
| **N stage, n (%)** |  |  | **0.046^*^** |
| N0 | 22(34.4%) | 34(53.1%) |  |
| N1 | 22(34.4%) | 16(25.0%) |  |
| N2 | 20(31.3%) | 14(21.9%) |  |
| **AJCC stage, n (%)** |  |  | **0.038^*^** |
| Stage1 | 10(15.6%) | 16(25.0%) |  |
| Stage2 | 12(18.8%) | 18(28.1%) |  |
| Stage3 | 42(65.6%) | 30(46.9%) |  |
| Tumor budding, n (%) |  |  | 0.343 |
| Negative | 9(14.0%) | 8(12.4%) |  |
| Weak | 31(48.4%) | 38(59.4%) |  |
| Moderate | 16(25.0%) | 15(23.4%) |  |
| Strong | 8(12.4%) | 3(4.6%) |  |
| Venous invasion, n (%) |  |  | 0.321 |
| Negative | 44(68.8%) | 49(76.6%) |  |
| Positive | 20(31.2%) | 15(23.4%) |  |
| Perineural invasion, n (%) |  |  | 0.720 |
| Negative | 36(28.1%) | 38(29.7%) |  |
| Positive | 28(21.9%) | 26(20.3%) |  |

Table S2. TLS features of involved colon cancer patients in validation cohort.

|  | LCC patients  (n=64) | RCC patients  (n=64) | *P* value |
| --- | --- | --- | --- |
| TLS number, mean ± SD | 5.64±4.627 | 18.06±9.106 | **<0.001** |
| Agg TLS number | 4.22±3.219 | 11.86±6.756 | **<0.001** |
| FL1 TLS number | 1.11±1.575 | 3.64±3.406 | **<0.001** |
| FL2 TLS number | 0.31±0.814 | 2.56±3.013 | **<0.001** |
| TLS density, mean ± SD | 4.07±3.894 | 9.67±5.537 | **<0.001** |
| TLS area density (%), mean ± SD | 0.49±0.381 | 1.05±1.133 | **<0.001** |
| Intra-tumoral TLS number, mean ± SD | 1.75±1.643 | 4.42±5.010 | **<0.001** |
| Peritumoral TLS number, mean ± SD | 3.89±3.805 | 13.61±7.195 | **<0.001** |
